# Ischemic preconditioning reduces the severity of ischemia-reperfusion injury of peripheral nerve in rats

**DOI:** 10.1186/1749-7221-1-2

**Published:** 2006-09-29

**Authors:** Yusuf Kenan Coban, Harun Ciralik, Ergul Belge Kurutas

**Affiliations:** 1 Dept. Of Plastic Surgery, Sutcuimam University, School of Medicine, Kahramanmaraş, Turkey; 2 Dept. Of Pathology, Sutcuimam University, School of Medicine, Kahramanmaraş, Turkey; 3 Dept. Of Biochemistry, Sutcuimam University, School of Medicine, Kahramanmaraş, Turkey

## Abstract

**Background and aim:**

Allow for protection of briefly ischemic tissues against the harmful effects of subsequent prolonged ischemia is a phenomennon called as Ischemic Preconditioning (IP). IP has not been studied in ischemia-reperfusion (I/R) model of peripheral nerve before. We aimed to study the effects of acute IP on I/R injury of peripheral nerve in rats.

**Method:**

70 adult male rats were randomly divided into 5 groups in part 1 experimentation and 3 groups in part 2 experimentation. A rat model of severe nerve ischemia which was produced by tying iliac arteries and all idenfiable anastomotic vessels with a silk suture (6-0) was used to study the effects of I/R and IP on nerve biochemistry. The suture technique used was a slip-knot technique for rapid release at time of reperfusion in the study. Cytoplasmic vacuolar degeneration was also histopathologically evaluated by light microscopic examination in sciatic nerves of rats at 7th day in part 2 study.

**Results:**

3 hours of Reperfusion resulted in an increase in nerve malondialdehyde levels when compared with ischemia and non-ischemia groups (p < 0.001 and p < 0.0001 respectively). IP had significantly lower nerve MDA levels than 3 h reperfusion group (p < 0.001). The differences between ischemic, IP and non-ischemic control groups were not significant (p > 0.05). There was also a significant decrease in vacoular degeneration of sciatic nerves in IP group than I/R group (p < 0.05).

**Conclusion:**

IP reduces the severity of I/R injury in peripheral nerve as shown by reduced tissue MDA levels at 3 th hour of reperfusion and axonal vacoulization at 7 th postischemic day.

## Background

Ischemia-reperfusion (I/R) causes oxidative injury and ischemic fiber degeneration (IFD), due to injury of the neuron and axon, after enough ischemic times, i.e.4–5 hours of peripheral nerve ischemia [1,2]. Maximal intercellular adhesion molecule-1 (ICAM-1) expression on endoneural vessels and polymorphonuclear monocytes reaches a peak at 24 th hour and macrophages increases nearly four fold at 48–72 hour of reperfusion after a 5 h of near-complete ischemia [3]. All these cells are responsible for demyelinisation and IFD at prolonged reperfusion after enough ischemic times in peripheral nerves [4,5]. Nerve lipid hydroperoxides reaches greatest levels at 3 hour and a gradual decline follows over the next month with reperfusion [6]. An aggrevated reperfusion injury in Streptozocine induced diabetic rats could be seen with less severe ischemic times [7]. Clinical experience related to I/R injury of peripheral nerve shows that neurologic recovery is possible, if reperfusion starts within 6 hours after ischemia [8].

Allow for protection of briefly ischemic tissues against the harmful effects of subsequent prolonged ischemia is a phenomennon called as Ischemic Preconditioning (IP)[9]. There are two distinct types of protection afforded by this adaptational reponse, i.e. acute and delayed preconditioning. The factors that initiate the acute and delayed preconditioning responses appear to be similiar. However, the protective effects of acute preconditioning are protein synthesis independent, while the effects of delayed preconditioning require protein synthesis. Adaptational responses to I/R injury have been demonstrated in different tissue types [10-14]. IP has not been studied in I/R model of peripheral nerve before. We aimed to study the effects of acute IP on I/R injury of peripheral nerve in rats.

## Materials and methods

### Animals

All animals were obtained from Experimental Research Laboratory of Sutcuimam University School of Medicine. The experimental design was approved by the Ethical commite of KSU. 200–250 g adult male spraque-dawley rats were used in the study. The animals were fed with standart rats diets until the surgical procedures.

We examined I/R induced pathological and biochemical changes along the lenght of scaitic nerve. Major arteries which supply rat hindlimb were occluded for 4 hours. Reperfusion was accomplished by the release of ties of abdominal aorta and its branches. Nerve pathology and biochemical analysis in sciatic nerve samples of the rats were assesed after 3 hours and 7 days of reperfusion. A total of 70 rats were used in the study. The study was divided into two part. Part 1 included the biochemical examination of Ischemia, I/R and I/R+IP on sciatic nerves of rats at the early period. Part 2 which consisted of 3 groups aimed to evaluate the histopathological changes in the nerves 7 days after the experimentation. The rats were randomly divided into following groups, 7 rats in each;

#### Part 1:Short time effects of I/R and IP

**Group I**- Normal adult male rats (Non-isch): Non-ischemic group, no intervention was made, simply sciatic nerve samples were taken.

**Group II- **Ischemic group (Ischemic control-0hR): 4 hours of limb ischemia were done and the samples were taken from the sciatic nerves after ischemic insult.

**Group III- **Ischemia-reperfusion group (3hR): 4 hours of ischemia and following 3 hours of reperfusion were done. After I/R of sciatic nerves samples were taken.

**Group IV**- I/R plus ischemic preconditioning group (3hR+IP): Preconditioning (three cycles of 5 minutes of short ischemia with 2 minute's intervals), and then 4 hours of ischemia with 3 hours of reperfusion.

#### Part 2: Long time effetcs of I/R and IP

**Group 1**- I/R with long duration (7dR):4 hours of ischemia and 7 days of reperfusion.

**Group 2**- Preconditioning plus I/R with long duration (7dR+IP): The same preconditioning protocol with the group IV, and then, 4 hours of ischemia with following 7 days of reperfusion. In both groups, sciatic nerve samples taken from both limb at 7th day were examined histopathologically.

**Group 3- **Sham operated group: Abdominal aorta and its collaterals were simply exposed under anesthesia, but no intervention was done. Then abdominal incision was primarly closed. At 7th day, sciatic nerve samples were taken as done in the other groups.

### Model of severe nerve ischemia

Our model of severe nerve ischemia was produced by tying of the iliolumbar and inferior mesenteric arteries followed by the temporary occlusion of the abdominal aorta and both iliac arteries [15]. We tied off all identifiable anastomotic vessels, including the iliolumbar and inferior mesenteric arteries. The aorta and iliac arteries were tied with a silk suture (6-0), using a slip-knot technique for rapid release, when needed. Measurements of the femoral blood pressure (BP) were used to monitor the completeness of the occlusion, and direct inspection of the sciatic nerve epineurial vessels showed that blood flow had been arrested. Sluggish flow was sometimes seen in these vessels several minutes after aortic occlusion, presumably due to partial reestablishment of anastomotic flow.

### Ischemia-reperfusion and ischemic preconditioning model

The rat was anesthetized with intraperitoneal pentobarbital (60 mg/kg) followed by surgery to produce IR. Ischemia was produced by ligating the abdominal aorta, the right iliac artery, the right femoral artery, and all identifiable collateral vessels supplying the sciatic nerves with 6-0 silk sutures. After 3 h of ischemia at 35°C, the ties were released using a slipknot technique for ready release and rapid reperfusion [16]. This procedure was done in IP groups for 3 times before the prolonged ischemia. Sciatic nerves were harvested at 3 hours and 1 week after ischemia surgery for the MDA measurements and histopathological studies, respectively.

### Neuropathology: edema and axonal vacoulisation

A sciatic nerve segment at 2 cm long was harvested from each animal. The sciatic nerves were osmicated, dehydrated, infiltrated, and embedded in Spurr's resin. Longitidunal sections of 1.0 cm were stained with hematoxylen eozine. Under 40× magnification, these sections were graded for edema and axonal vacoulisation using previously described methods [17]. The axon may be swollen or shrunken, watery and light, or dark and shrunken. Secondary myelin changes were typically seen, including attenuation, collapse, or break-down. For each section, the vacoulisation and edema were semi-quantitatively graded from 0 to 3 as follows: 0-normal, 1-mild, 2-moderate and 3-severe. No distinction was made as to endoneurial, perivascular, or subperineurial edema. A mean value for each rat was calculated after examination of four sections represented that case.

### Statistics

The values were expressed as mean ± standart of deviation. The differences between the groups were analysed by using ANOVA. Non-parametric data was evaluated by Mann Whithney-U test. A p value less than 0.05 was considered as significant.

## Results

### MDA levels in sciatic nerve

During the occlusion of aorta and iliac arteries, the measurements of femoral blood pressure aproximated to zero values in rats of all the groups.

**Figure 1:**
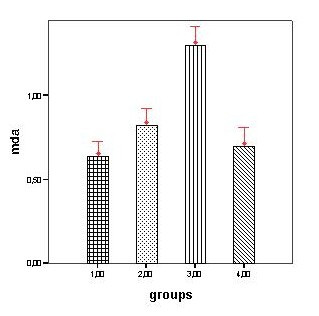
Sciatic nerve MDA (nmol/mg protein) levels in groups. (1.00:non-ischemic controls, 2.00:ischemic preconditioning, 3.00:ischemia-3 h reperfusion, 4.00:ischemia only). Bars show means. Error bars show 95.0% CI of means.

The MDA levels of nerve tissue segments in different groups and nerve vacuolisation degrees is shown in the table 1. Ischemic preconditioning group had significantly lower nerve MDA levels than reperfusion group (p < 0.001). The differences between ischemic, IP and non-ischemic control groups were not significant (p > 0.05), (figure 1).

**Table 1:** Non-parametric evaluation scores in part 2 experimentation.

*Parameter*	*I/R*	*IP*	*Sham*
**Edema**	3	2*	0**
**Vacoulisation**	2	1	0

The scale for the evaluated parameters as follows: normal-0, mild-1, moderate-2, severe-3 for endoneurial edema;no vacoulisation-0, mild vacoulisation-1, massive vacoulisation-2 for axonal vacuolisation.(* p < 0.05 for I/R versus IP, **p < 0.00001 for I/R versus IP).

### Histopathologic changes

**Figure 2:**
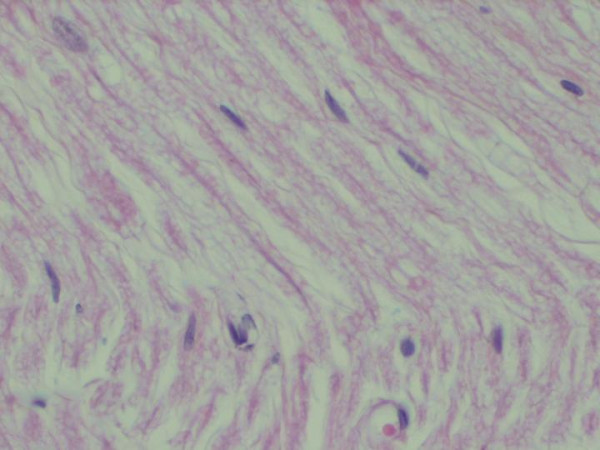
Normal architechture of sciatic nerve of rat is seen (sham group), Hematoxylen esozine 40× magnification.

**Figure 3:**
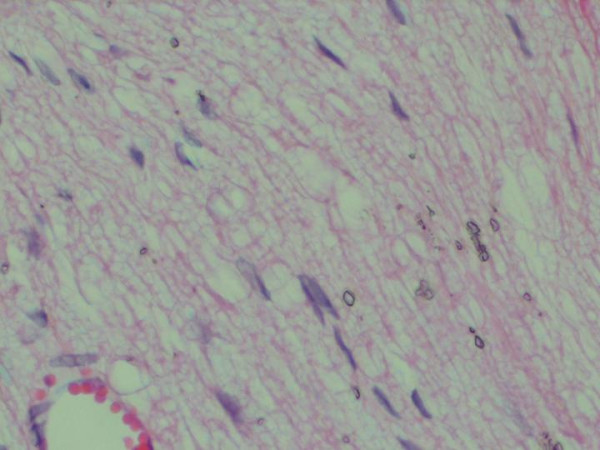
Increased axonal vacuolisation degeneration is seen at longitidunal section of sciatic nerve (I/R group, score 3), Hematoxylen esozine 40× magnification.

**Figure 4:**
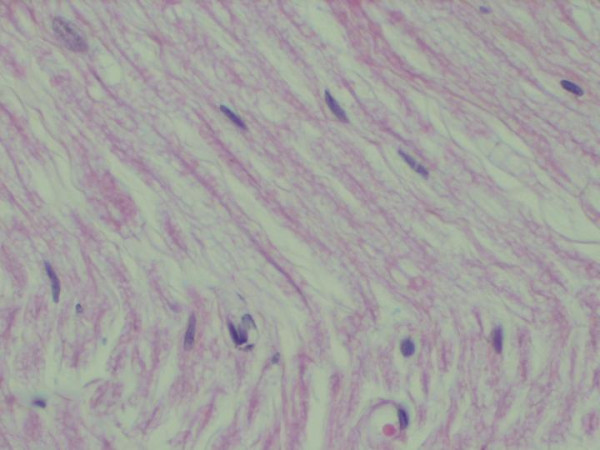
Mild vacuolisation in axons of sciatic nerve (ischemic preconditioning group, score 2), Hematoxylen esozine 40× magnification.

Great cytoplasmic vacoulisation caused by proliferation and dilation of the rough and smooth endoplasmic reticulum and golgi apparatus was observed in I/R and IP groups of part 2 experimentation. The intramyelinic edema within nerve fibers was seen not only in perivascular region, but also, in endoneurial vessels (Figures 2, 3 and 4). IP group had a significantly good histopathologic score than I/R group (p < 0.05). Table 1 shows the scores in the groups.

## Discussion

Nerve pathology in acute ischemic injury has beeen delineated in peripheral nerve and reperfusion injury could amplify ischemic pathology. Nerve ischemia plays a major role in the development of pathological alteration in varous neuropathies and the effects of ischemia are amplified by reperfusion in various tissues. In nerve tissue, two types of edema is described after I/R; endoneural edema and intramyelinic edema [17]. Endoneural edema reflects in blood-nerve barrier and possibly reflects endoneural events especially severity of IFD. Myelin appears to be particularly susceptible to activated free radicals, activated neutrophils and cytokine formation. Severe ischemia to nerve results in energy rundown followed by conduction failure and fiber degeneration. Inflammatory responses to IR have not only been confined to a few days (up to 7–14 days) of reperfusion, but also much more extended time (up to 42 days) of reperfusion [18]. Between 7 days and 14 days of reperfusion, the IFD was reported to be the most prominent. Morphological changes of IFD at the light microscopic level occur in concert with endoneurial edema at the 7 and 14 day reperfusion time-points. I/R injury of sciatic nerve has been shown to increase proinlammatory cytokines which are primarly responsible demyelinization after reperfusion in peripheral nerves [19]. Another important indicator of I/R injury of peripheral nerve is Nitric Oxide products which were found as increased at the first 24 hours of reperfusion in nerve tissue and their elevation has been reported to play an essential role in reducing the severity of the I/R injury by inhibiting neutrophil adhesion in postcapillary venules and by decreasing microvascular constriction [20]. In our study, axonal changes at 7 th day were evaluated. It has been seen that IP treated group showed less cytoplasmic vacuolisation and edema formation than I/R group (p < 0.05). This finding was concominant to the finding of decreased oxidative injury (i.e. decreased MDA levels in nerve tissue) seen in IP pretreated group.

Previously protective effects of IP in intestine, liver, myocard, skeletal muscle and pancreas tissues has been shown [10-14,21,22]. What play role in the protective effect of IP is not exactly known, but some putative mechanims, which are mostly dealed with countering the proinflammatory and proapoptotic effects generated during IR have been put forward [23]. IP has been shown to decrease the formation of hydroxyl radicals during reperfusion [24]. A reduced TNF-alpha and ICAM-1 mRNA expression seen after IP may account for the inhibitory effects of IP on leukocyte adhesion and ameloriated microcirculatory disturbance after IR in vivo [23-26].

The protective effects of IP against lesions caused by subsequent severe ischemia was primarly described in the heart by Murry et al [9]. Increased antioxidant enzyme activities which may be an indirect indicator of the reduced injury after I/R has been shown in brain ischemic tolerance by IP [27]. However to best of our knowledge, nobody has studied this phenomennon in peripheral somatic nerve. The benefical effect of IP in rat sciatic nerve was manifested by a reduction in MDA tissue levels at 3th hour of reperfusion and ischemic fiber degeneration (IFD) at 7 th postischemic day of reperfusion, in the present study. Lida et al. identified pathologically three phases as follows: phase 1-early reperfusion minimal edema; phase-2 7 th and 14 th day of reperfusion prominent fiber degeneration and endoneurial edema; phase-3 28 th and 42 th days abundant small regenerating fiber clusters, minimal edema [18]. Our observation period is limited to up 7th day, i.e. phase-1. To best of our knowledge, this is the first semiquantitative study that shows an decreased IFD after IR due to the pretreatment with IP. Further studies are needed for understanding that IP may have strategic role in treatment of I/R related peripheral nerve injuries.
